# (*E*)-4-(2,3-Dihydro-1,3-benzothia­zol-2-yl­idene)-3-methyl-1-phenyl-1*H*-pyrazol-5(4*H*)-one

**DOI:** 10.1107/S1600536810013176

**Published:** 2010-04-17

**Authors:** Imane Chakibe, Abdelfettah Zerzouf, El Mokhtar Essassi, Martin Reichelt, Hans Reuter

**Affiliations:** a Laboratoire de Chimie Organique et Études Physicochimiques, ENS Rabat, Morocco; b Laboratoire de Chimie Organique Hétérocyclique, Université Mohammed V Rabat, Morocco; c Institute of Nanomaterials and Nanotechnology, Avenue Armées Royals, Rabat, Morocco; d Institute of Chemistry, University of Osnabrück, Barbarastrasse 7, D-49069 Osnabrück, Germany

## Abstract

In the title compound, C_17_H_13_N_3_OS, the dihedral angle between the ring systems is 2.22 (5)°. The N—H grouping participates in both intra- and intermolecular N—H⋯O hydrogen bonds, the latter leading to dimers related by a twofold rotation axis.

## Related literature

For related structures, see: Teo *et al.* (1993 ▶); Chen (1994 ▶); Sawusch & Schilde (1999 ▶); Chu *et al.* (2003 ▶); Liu *et al.* (2004 ▶). For related literature, see: Harnden *et al.* (1978 ▶); Hatheway *et al.* (1978 ▶); Londershausen (1996 ▶); Tewari & Mishra (2001 ▶).
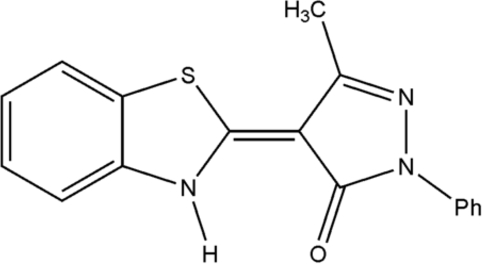


## Experimental

### 

#### Crystal data

C_17_H_13_N_3_OS
*M* *_r_* = 307.36
Monoclinic, 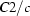

*a* = 27.0144 (8) Å
*b* = 7.4021 (2) Å
*c* = 14.0523 (4) Å
β = 97.466 (1)°
*V* = 2786.12 (14) Å^3^
*Z* = 8
Mo *K*α radiation
μ = 0.24 mm^−1^
*T* = 100 K
0.40 × 0.28 × 0.20 mm

#### Data collection

Bruker APEXII with a CCD detector diffractometer
Absorption correction: multi-scan (*SADABS*; Bruker, 2008 ▶) *T*_min_ = 0.875, *T*_max_ = 0.956
57380 measured reflections
3359 independent reflections
2928 reflections with *I* > 2σ(*I*)
*R*_int_ = 0.041

#### Refinement

*R*[*F* ^2^ > 2σ(*F* ^2^)] = 0.030
*wR*(*F* ^2^) = 0.082
*S* = 1.05
3359 reflections
203 parameters
H-atom parameters constrained
Δρ_max_ = 0.37 e Å^−3^
Δρ_min_ = −0.26 e Å^−3^

### 

Data collection: *APEX2* (Bruker, 2008 ▶); cell refinement: *SAINT* (Bruker, 2008 ▶); data reduction: *SAINT*; program(s) used to solve structure: *SHELXS97* (Sheldrick, 2008 ▶); program(s) used to refine structure: *SHELXL97* (Sheldrick, 2008 ▶); molecular graphics: *DIAMOND* (Brandenburg, 2006 ▶); software used to prepare material for publication: *SHELXTL* (Sheldrick, 2008 ▶).

## Supplementary Material

Crystal structure: contains datablocks I, global. DOI: 10.1107/S1600536810013176/fk2016sup1.cif

Structure factors: contains datablocks I. DOI: 10.1107/S1600536810013176/fk2016Isup2.hkl

Additional supplementary materials:  crystallographic information; 3D view; checkCIF report

## Figures and Tables

**Table 1:** Hydrogen-bond geometry (Å, °)

*D*—H⋯*A*	*D*—H	H⋯*A*	*D*⋯*A*	*D*—H⋯*A*
N3—H3⋯O15	0.88	2.24	2.8483 (14)	126
N3—H3⋯O15^i^	0.88	2.22	2.9161 (13)	136

Symmetry code: (i) 
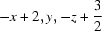
.

**Fig. 1.**
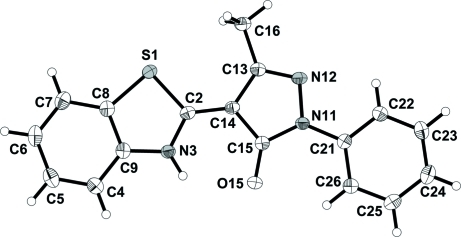
Ball-and-stick model of 3 with the atomic numbering scheme used; with exception of the hydrogen atoms, which are shown as spheres with common isotropic radius, all other atoms are represented as thermal displacement ellipsoids showing the 50% probability level of the corresponding atom.

**Fig. 2.**
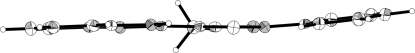
Side view of the ball-and-stick model of 3 showing slightly curved conformation of the different ring systems.

**Fig. 3.**
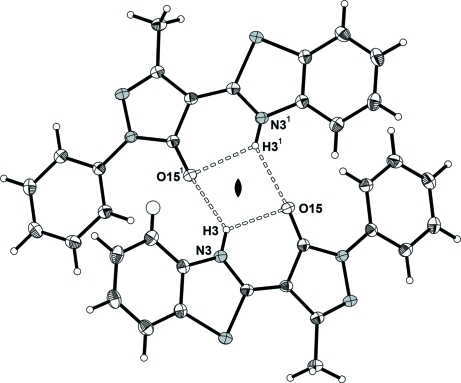
Hydrogen bonded dimers of 3 with the intra- and intermolecular bifurcated hydrogen bonds indicated as broken lines; symmetry code 1) -x+2, y, -z+3/2.

**Fig. 4.**
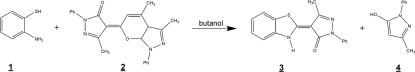
The formation of the title compound.

## supplementary crystallographic information

### Comment

The chemistry of pyrazole derivatives has been the subject of much interest due to their importance for various applications and their widespread potential and proven biological and pharmacological activities such as anti-inflammatory (Tewari *et al.*, 2001), antimicrobial, antiviral (Harnden *et al.*, 1978), anti-tumor (Hatheway *et al.*, 1978), anti-fungal and pesticidal substances (Londershausen *et al.*, 1996).

The new pyrazole derivative (*E*)-4-(benzo[*d*]thiazol-2-(3*H*)-ylidene)-3-methyl-1- phenyl-1*H*-pyrazol-5(*4H*)-one, 3, was synthesised according to reaction scheme 1. The solid state structure of 3 (Fig. 1) shows the typical structural features of its three subunits: a phenyl rest, a pyrazole derivative and a benzothiazole-like fragment.

In the pyrazol moiety the shortening of the bond between N12 and C13 [1.307 (2) Å] corresponds to a double bond in accordance with the formulation of the double bond in scheme 1.

All of the three subunits are for themselves planar, deviations from the least-square planes are small. Within the benzo[d]thiazole the greatest deviations result from the fact that the phenyl ring is planar whereas the remaining atoms of the thiazole ring are out of this plane with a maximum at C2 [-0.055 (2) Å]. Moreover, the complete molecule adopts are slightly curved conformation (Fig. 2). In the case of the 1,3-benzothiazole-pyrazole bond planarity should be result from the double bond [*d*(C—C) = 1.388 (2) Å] between the two subunits. Nevertheless, the interplanar dihedral angle between both ring systems is 2.22 (5)°. Between the pyrazole and the phenyl fragment the carbon-carbon single bond is shortened [*d*(C—C) = 1.416 (2) Å] but even longer than a double bond. In this case, the interplanar dihedral angle between both fragments is 7.16 (6)°. Similar values between these two subunits were obsereved earlier (Liu *et al.* 2004).

In the solid state, 3 forms dimers (Fig. 3) by bifurcated hydrogen bonds between the NH-group [N3] of the 1,3-benzothiazole-rest and O15 of a neighbouring molecule, both related by a twofold rotation axis. The second part of the bifurcated hydrogen bond system combines the NH-group with O15 within the same molecule.

### Experimental

1.98 g (0.1 mol) (*E*)-3-methyl-4-(4-methyl-1-phenylpyrano[2,3-*c*]pyrazol-6(1*H*)-ylidene)-1-phenyl-1*H*-pyrazol-5(4*H*)-one, 2, were refluxed for 72 h with 2.136 ml (0.02 mol) 2-aminobenzenethiol, 1, in 50 ml n-butanol. On cooling the product forms transparent colourless crystals, which were filtered off in a yield of 50%. A suitable single crystal was selected under a polarization microsope and mounted on a 50 µm MicroMesh MiTeGen Micromount^TM ^using FROMBLIN Y perfluoropolyether (LVAC 16/6, Aldrich).

### Refinement

Hydrogen atoms were clearly identified in difference Fourier syntheses, idealized and refined at calculated positions riding on the carbon atoms with C—H = 0.98 Å for methyl H atoms, 0.95 Å for aromatic H atoms and N—H = 0.88 Å. Methyl groups were allowed to rotate around the C—C–bond. Three common isotropic displacement parameters for the H–atoms of the three different subunits were refined.

### Crystal data

**Table:**

C_17_H_13_N_3_OS	*F*(000) = 1280
*M**_r_* = 307.36	*D*_x_ = 1.466 Mg m^−^^3^
Monoclinic, *C*2/*c*	Mo *K*α radiation, λ = 0.71073 Å
Hall symbol: -C 2yc	Cell parameters from 9789 reflections
*a* = 27.0144 (8) Å	θ = 2.9–30.6°
*b* = 7.4021 (2) Å	µ = 0.24 mm^−^^1^
*c* = 14.0523 (4) Å	*T* = 100 K
β = 97.466 (1)°	Prism, red
*V* = 2786.12 (14) Å^3^	0.40 × 0.28 × 0.20 mm
*Z* = 8	

### Data collection

**Table:**

Bruker APEXII with a CCD detector diffractometer	3359 independent reflections
Radiation source: fine-focus sealed tube	2928 reflections with *I* > 2σ(*I*)
graphite	*R*_int_ = 0.041
φ and ω scans	θ_max_ = 28.0°, θ_min_ = 2.9°
Absorption correction: multi-scan (*SADABS*; Bruker, 2008)	*h* = −35→35
*T*_min_ = 0.875, *T*_max_ = 0.956	*k* = −9→9
57380 measured reflections	*l* = −18→18

### Refinement

**Table:**

Refinement on *F*^2^	Primary atom site location: structure-invariant direct methods
Least-squares matrix: full	Secondary atom site location: difference Fourier map
*R*[*F*^2^ > 2σ(*F*^2^)] = 0.030	Hydrogen site location: difference Fourier map
*wR*(*F*^2^) = 0.082	H-atom parameters constrained
*S* = 1.05	*w* = 1/[σ^2^(*F*_o_^2^) + (0.0331*P*)^2^ + 3.8168*P*] where *P* = (*F*_o_^2^ + 2*F*_c_^2^)/3
3359 reflections	(Δ/σ)_max_ = 0.001
203 parameters	Δρ_max_ = 0.37 e Å^−^^3^
0 restraints	Δρ_min_ = −0.26 e Å^−^^3^

### Special details

**Table:**

Geometry. All esds (except the esd in the dihedral angle between two l.s. planes) are estimated using the full covariance matrix. The cell esds are taken into account individually in the estimation of esds in distances, angles and torsion angles; correlations between esds in cell parameters are only used when they are defined by crystal symmetry. An approximate (isotropic) treatment of cell esds is used for estimating esds involving l.s. planes.
Refinement. Refinement of *F*^2^ against ALL reflections. The weighted *R*-factor wR and goodness of fit *S* are based on *F*^2^, conventional *R*-factors *R* are based on *F*, with *F* set to zero for negative *F*^2^. The threshold expression of *F*^2^ > σ(*F*^2^) is used only for calculating *R*-factors(gt) etc. and is not relevant to the choice of reflections for refinement. *R*-factors based on *F*^2^ are statistically about twice as large as those based on *F*, and *R*- factors based on ALL data will be even larger.

### Fractional atomic coordinates and isotropic or equivalent isotropic displacement parameters (Å^2^)

**Table:**

	*x*	*y*	*z*	*U*_iso_*/*U*_eq_	
S1	1.059848 (12)	0.17433 (4)	1.08323 (2)	0.01678 (9)	
C2	1.02064 (5)	0.21310 (17)	0.97688 (9)	0.0151 (2)	
N3	1.04276 (4)	0.16259 (14)	0.90040 (7)	0.0160 (2)	
H3	1.0281	0.1754	0.8411	0.0247 (19)*	
C4	1.12040 (5)	0.02162 (18)	0.85676 (10)	0.0198 (3)	
H4	1.1098	0.0244	0.7896	0.0247 (19)*	
C5	1.16636 (5)	−0.04948 (19)	0.89339 (11)	0.0235 (3)	
H5	1.1876	−0.0965	0.8506	0.0247 (19)*	
C6	1.18205 (5)	−0.05341 (19)	0.99209 (11)	0.0250 (3)	
H6	1.2138	−0.1025	1.0152	0.0247 (19)*	
C7	1.15201 (5)	0.01318 (19)	1.05696 (10)	0.0222 (3)	
H7	1.1626	0.0104	1.1241	0.0247 (19)*	
C8	1.10583 (5)	0.08405 (17)	1.02029 (9)	0.0174 (2)	
C9	1.09021 (5)	0.08893 (17)	0.92167 (9)	0.0167 (2)	
N11	0.89806 (4)	0.38442 (15)	0.90615 (7)	0.0151 (2)	
N12	0.90079 (4)	0.40940 (15)	1.00569 (7)	0.0167 (2)	
C13	0.94462 (5)	0.34924 (16)	1.04238 (9)	0.0157 (2)	
C14	0.97279 (5)	0.28461 (17)	0.96994 (8)	0.0150 (2)	
C15	0.94124 (5)	0.30754 (16)	0.87995 (9)	0.0147 (2)	
O15	0.94948 (3)	0.26778 (13)	0.79710 (6)	0.01790 (19)	
C16	0.95996 (5)	0.35330 (18)	1.14808 (9)	0.0194 (3)	
H16A	0.9322	0.3982	1.1800	0.030 (3)*	
H16B	0.9888	0.4333	1.1628	0.030 (3)*	
H16C	0.9689	0.2310	1.1710	0.030 (3)*	
C21	0.85536 (5)	0.45211 (17)	0.84799 (9)	0.0154 (2)	
C22	0.81913 (5)	0.54324 (19)	0.89193 (9)	0.0201 (3)	
H22	0.8227	0.5545	0.9598	0.027 (2)*	
C23	0.77785 (5)	0.61730 (19)	0.83640 (10)	0.0219 (3)	
H23	0.7534	0.6799	0.8666	0.027 (2)*	
C24	0.77185 (5)	0.60084 (19)	0.73731 (10)	0.0224 (3)	
H24	0.7436	0.6522	0.6995	0.027 (2)*	
C25	0.80761 (5)	0.50843 (19)	0.69414 (10)	0.0225 (3)	
H25	0.8035	0.4960	0.6263	0.027 (2)*	
C26	0.84937 (5)	0.43350 (18)	0.74838 (9)	0.0186 (3)	
H26	0.8736	0.3703	0.7179	0.027 (2)*	

### Atomic displacement parameters (Å^2^)

**Table:**

	*U*^11^	*U*^22^	*U*^33^	*U*^12^	*U*^13^	*U*^23^
S1	0.01820 (16)	0.01755 (16)	0.01406 (15)	0.00014 (12)	0.00009 (11)	0.00048 (11)
C2	0.0180 (6)	0.0128 (5)	0.0142 (5)	−0.0027 (4)	0.0010 (4)	0.0002 (4)
N3	0.0175 (5)	0.0166 (5)	0.0138 (5)	0.0006 (4)	0.0019 (4)	0.0002 (4)
C4	0.0221 (6)	0.0161 (6)	0.0222 (6)	−0.0004 (5)	0.0062 (5)	0.0012 (5)
C5	0.0213 (7)	0.0184 (6)	0.0322 (7)	0.0009 (5)	0.0090 (5)	0.0006 (5)
C6	0.0175 (6)	0.0221 (7)	0.0348 (8)	0.0030 (5)	0.0009 (5)	0.0030 (6)
C7	0.0211 (6)	0.0196 (6)	0.0248 (7)	−0.0003 (5)	−0.0013 (5)	0.0025 (5)
C8	0.0175 (6)	0.0142 (6)	0.0206 (6)	−0.0015 (5)	0.0026 (5)	0.0007 (5)
C9	0.0168 (6)	0.0133 (6)	0.0200 (6)	−0.0014 (5)	0.0024 (5)	0.0019 (5)
N11	0.0169 (5)	0.0162 (5)	0.0123 (5)	0.0007 (4)	0.0025 (4)	−0.0001 (4)
N12	0.0204 (5)	0.0177 (5)	0.0122 (5)	0.0000 (4)	0.0026 (4)	−0.0010 (4)
C13	0.0192 (6)	0.0132 (6)	0.0150 (6)	−0.0018 (5)	0.0031 (5)	−0.0006 (4)
C14	0.0177 (6)	0.0138 (6)	0.0135 (5)	−0.0016 (5)	0.0016 (4)	−0.0003 (4)
C15	0.0157 (6)	0.0129 (5)	0.0157 (6)	−0.0021 (4)	0.0024 (4)	0.0004 (4)
O15	0.0188 (4)	0.0220 (5)	0.0132 (4)	0.0011 (4)	0.0034 (3)	−0.0014 (3)
C16	0.0227 (6)	0.0208 (6)	0.0146 (6)	−0.0001 (5)	0.0017 (5)	−0.0014 (5)
C21	0.0153 (6)	0.0131 (5)	0.0176 (6)	−0.0027 (5)	0.0015 (4)	0.0014 (4)
C22	0.0197 (6)	0.0226 (7)	0.0182 (6)	0.0011 (5)	0.0037 (5)	−0.0001 (5)
C23	0.0175 (6)	0.0212 (6)	0.0275 (7)	0.0011 (5)	0.0050 (5)	0.0003 (5)
C24	0.0177 (6)	0.0209 (7)	0.0271 (7)	−0.0007 (5)	−0.0021 (5)	0.0038 (5)
C25	0.0238 (7)	0.0245 (7)	0.0183 (6)	−0.0010 (5)	−0.0008 (5)	0.0009 (5)
C26	0.0195 (6)	0.0186 (6)	0.0178 (6)	−0.0004 (5)	0.0025 (5)	−0.0003 (5)

### Geometric parameters (Å, °)

**Table:**

S1—C2	1.7397 (13)	N12—C13	1.3067 (16)
S1—C8	1.7485 (13)	C13—C14	1.4303 (17)
C2—N3	1.3488 (16)	C13—C16	1.4896 (17)
C2—C14	1.3883 (18)	C14—C15	1.4402 (16)
N3—C9	1.3892 (16)	C15—O15	1.2486 (15)
N3—H3	0.8800	C16—H16A	0.9800
C4—C5	1.3845 (19)	C16—H16B	0.9800
C4—C9	1.3924 (18)	C16—H16C	0.9800
C4—H4	0.9500	C21—C26	1.3949 (17)
C5—C6	1.397 (2)	C21—C22	1.3969 (18)
C5—H5	0.9500	C22—C23	1.3882 (19)
C6—C7	1.387 (2)	C22—H22	0.9500
C6—H6	0.9500	C23—C24	1.3861 (19)
C7—C8	1.3892 (18)	C23—H23	0.9500
C7—H7	0.9500	C24—C25	1.386 (2)
C8—C9	1.3956 (17)	C24—H24	0.9500
N11—C15	1.3896 (16)	C25—C26	1.3921 (18)
N11—N12	1.4034 (14)	C25—H25	0.9500
N11—C21	1.4158 (16)	C26—H26	0.9500
			
C2—S1—C8	91.25 (6)	C14—C13—C16	127.71 (12)
N3—C2—C14	123.68 (11)	C2—C14—C13	130.85 (12)
N3—C2—S1	110.81 (9)	C2—C14—C15	123.10 (11)
C14—C2—S1	125.50 (10)	C13—C14—C15	106.05 (11)
C2—N3—C9	115.43 (10)	O15—C15—N11	126.94 (11)
C2—N3—H3	122.3	O15—C15—C14	129.34 (12)
C9—N3—H3	122.3	N11—C15—C14	103.72 (10)
C5—C4—C9	117.76 (12)	C13—C16—H16A	109.5
C5—C4—H4	121.1	C13—C16—H16B	109.5
C9—C4—H4	121.1	H16A—C16—H16B	109.5
C4—C5—C6	121.25 (13)	C13—C16—H16C	109.5
C4—C5—H5	119.4	H16A—C16—H16C	109.5
C6—C5—H5	119.4	H16B—C16—H16C	109.5
C7—C6—C5	121.13 (13)	C26—C21—C22	119.65 (12)
C7—C6—H6	119.4	C26—C21—N11	121.59 (11)
C5—C6—H6	119.4	C22—C21—N11	118.74 (11)
C6—C7—C8	117.66 (13)	C23—C22—C21	120.00 (12)
C6—C7—H7	121.2	C23—C22—H22	120.0
C8—C7—H7	121.2	C21—C22—H22	120.0
C7—C8—C9	121.27 (12)	C24—C23—C22	120.70 (13)
C7—C8—S1	128.27 (11)	C24—C23—H23	119.7
C9—C8—S1	110.45 (10)	C22—C23—H23	119.7
N3—C9—C4	127.02 (12)	C23—C24—C25	119.07 (12)
N3—C9—C8	112.04 (11)	C23—C24—H24	120.5
C4—C9—C8	120.93 (12)	C25—C24—H24	120.5
C15—N11—N12	112.38 (10)	C24—C25—C26	121.21 (12)
C15—N11—C21	129.86 (10)	C24—C25—H25	119.4
N12—N11—C21	117.51 (10)	C26—C25—H25	119.4
C13—N12—N11	106.02 (10)	C25—C26—C21	119.36 (12)
N12—C13—C14	111.83 (11)	C25—C26—H26	120.3
N12—C13—C16	120.47 (11)	C21—C26—H26	120.3

### Hydrogen-bond geometry (Å, °)

**Table:**

*D*—H···*A*	*D*—H	H···*A*	*D*···*A*	*D*—H···*A*
N3—H3···O15	0.88	2.24	2.8483 (14)	126
N3—H3···O15^i^	0.88	2.22	2.9161 (13)	136

Symmetry codes: (i) −*x*+2, *y*, −*z*+3/2.
